# Microbiotoxicity: an under-recognised player in drug efficacy, toxicity, and health outcomes

**DOI:** 10.1038/s44259-025-00165-5

**Published:** 2025-12-23

**Authors:** Shirley Do Nascimento, Anastasia A. Theodosiou, Chrysi Sergaki

**Affiliations:** 1 Science Research and Innovation, Medicines and Healthcare products Regulatory Agency, Blanche Lane, South Mimms, Potters Bar, Hertfordshire, UK; 2 School of Infection & Immunity, University of Glasgow, Glasgow, UK

**Keywords:** Policy and public health in microbiology, Health policy, Disease prevention

## Abstract

The gut microbiome regulates immunity, inflammation, and metabolism. Disruption by antibiotic and non-antibiotic drugs, termed microbiotoxicity, may impair efficacy of treatments, including cancer immunotherapy and vaccination, and contribute to antimicrobial resistance (AMR). This review explores microbiotoxicity’s clinical impacts, highlighting non-antibiotic drug effects. Further research into drug-microbiome interactions in future may help inform prescribing practices and drug development as a way to improve health outcomes, reduce toxicity, and support AMR stewardship.

## Introduction

The gut microbiome plays vital roles in supporting immune function, metabolism, and controlling inflammation^1^. This complex microbial ecosystem harbours bacteria possessing antimicrobial resistance (AMR) genes that can spread through horizontal gene transfer – a process that is exacerbated by the use of antibiotics^1^. Antibiotic use disrupts the microbiome balance, reducing diversity and microbiome resilience, and enabling resistant strains to thrive^1^. In addition to altering microbial composition, antibiotics can also impair the gut microbiota’s metabolism and functions^1^. These changes are associated with altered immune function and greater host susceptibility to diseases^2^. Moreover, antibiotics affect not only the gut microbiome, but also oral, respiratory, skin, and vaginal microbiota, with implications for host health^3^.

**Fig. 1:**
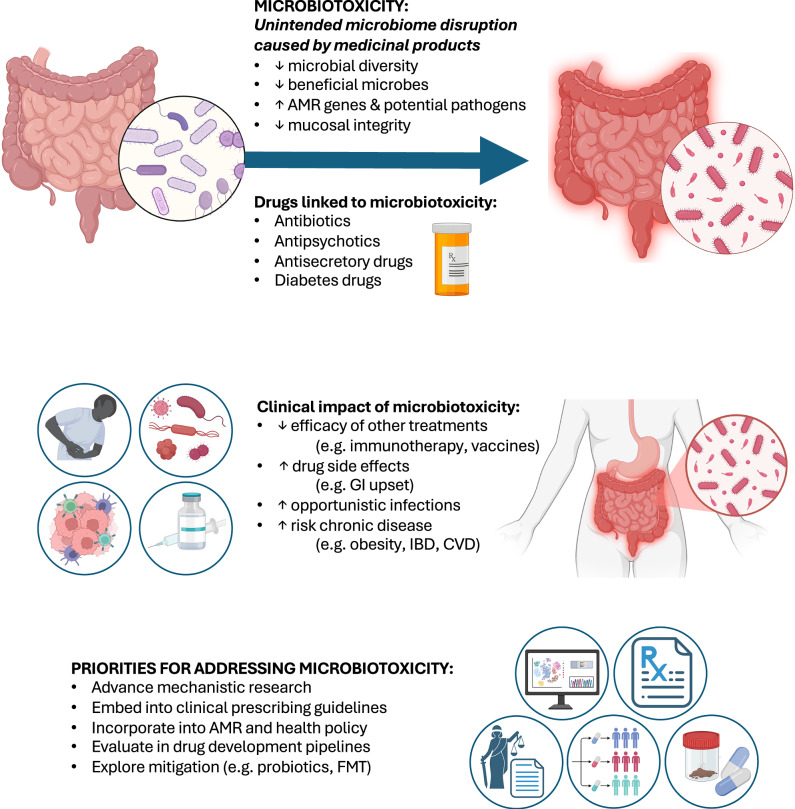
Microbiotoxicity causes, consequences, and strategic priorities to enhance patient outcomes. AMR antimicrobial resistance, CVD cardiovascular disease, FMT faecal microbiota transplant, GI gastrointestinal, IBD inflammatory bowel disease. Figure created with BioRender.com.

The extent to which microbiome considerations are incorporated in prescribing practices, antimicrobial resistance stewardship programmes, and wider health policy varies. Microbiotoxicity describes the unintended microbiome disruption caused by agents with antimicrobial properties, including antibiotics, other medicinal drugs, and non-pharmacological consumer, dietary and agricultural products (see Box 1)^4,5^. This paper reviews recent evidence relating to gut microbiotoxicity due to drug usage to underline the microbiome’s critical role in drug metabolism and health. In addition, this paper highlights research on the role of microbiotoxicity during unlinked interventions, including cancer immunotherapy and bone marrow transplantation (see Fig. 1). Such evidence suggests that microbiotoxicity may have broader medical and economic implications than previously appreciated.

Box 1. Definitions and evolution of microbiotoxicity Theodosiou et al. introduced the term microbiotoxicity to highlight the unintended, harmful effects of antibiotics on the patient’s microbiome, urging clinicians to balance therapeutic benefits with potential microbiome-negative effects^4^. Unlike dysbiosis or other descriptors of microbiome state, microbiotoxicity refers to an agent-specific property, analogous to nephrotoxicity or hepatotoxicity, denoting the capacity of a drug or compound to disrupt the microbiome’s structure or function^4^. This concept, originally coined to describe the effect of antibiotics on the microbiome, has since expanded to include non-antibiotic drugs (e.g., statins, nonsteroidal anti-inflammatory drugs, metformin), food additives (e.g., emulsifiers, sweeteners), and biocidal consumer products (e.g., antiseptics, silver nanoparticles), reflecting a One Health Perspective^5^. With this addition, the authors proposed a formal recognition of the microbiome as a human organ system and for its inclusion in drug development and clinical decision-making. Although clinically validated tests of microbiome health are still in development, research biomarkers, such as diversity measures, metabolite profiles, and strain-level signatures, are rapidly advancing^62,63^. These developments suggest that assessing microbiotoxicity in drug development and toxicology is feasible, with potential applications in routine patient care.

## Antibiotic use, microbiome perturbation, and health outcomes

Antibiotics are amongst the most frequently prescribed drugs, used to treat or prevent bacterial infections^6^. However, both short- and long-term antibiotic use have been linked to microbiotoxicity, including loss of microbiome resilience, reduced mucosal integrity and altered immune function, which are associated with downstream adverse health outcomes^4,6^. Microbiotoxicity encompasses both the disruption of microbial communities and their function, but also the selection and persistence of AMR genes within the microbiome, increasing the risk of resistance spreading to pathogenic bacteria. Recognising microbiotoxicity as a contributor to the emergence and spread of AMR highlights the need for integrated stewardship strategies that consider both the direct and indirect consequences of drug exposure on the microbiome^7^. For example, a seven-day course of azithromycin, frequently prescribed to treat chest, throat, and nose infections, significantly decreased the keystone anaerobes *Bifidobacteria* during treatment, with a higher abundance of *Bacteroides* in the gut up to six months later, and a rise in gut AMR genes^8,9^. These changes in bacterial relative abundance are associated with conditions like irritable bowel syndrome and obesity^10^. In another study, a seven-day course of amoxicillin was found to reduce the relative abundance of *Blautia* species, which are essential for regulating gut lining integrity and inflammation, with effects persisting up to six months^8,11^. Even in healthy subjects, a seven-day course of amoxicillin and clavulanate led to an overgrowth of *E. coli* in the gut and antibiotic-associated diarrhoea^12^.

Furthermore, studies are beginning to show that the effect of antibiotics on the microbiome may not fully return to its original state even many months after antibiotics^8,11,13^. For example, participants prescribed metronidazole to treat *Helicobacter pylori* infection *–* a cause of peptic ulcers and a risk factor for gastric cancer *–* experienced ongoing microbiome disruption one year later^14^. By contrast, participants who were randomised to treatments without metronidazole experienced full microbiome recovery by eight weeks^14^.

Reframing these symptoms as possible manifestations of microbiotoxicity reflects the reported associations of antibiotics on patients’ immune and gastrointestinal systems. This approach is consistent with studies linking the systemic health problems associated with microbiotoxicity, such as mood disorders and allergic sensitisation^10^. Understanding this interplay may inform research aimed at improving health outcomes by prioritising new treatment strategies that minimise side effects whilst maximising clinical efficacy.

## Impact of microbiotoxicity on subsequent medical interventions

Recent evidence suggests that the microbiome may modulate host response to interventions such as cancer immunotherapy and vaccines. Thus, antibiotics that alter gut microbiome composition may impact the efficacy and toxicity of subsequent treatments. Routy et al. demonstrated that cancer patients who received antibiotics one or two months before immune checkpoint inhibitor (ICI) treatment had a significantly shorter overall survival and progression-free survival (*p* < 0.05), concluding that antibiotic use is an indicator of reduced immunotherapy treatment efficacy, independent of other prognostic factors^15^. Additional studies show that even concomitant use of antibiotics with ICI therapies may lead to shorter survival rates^16–21^. Similarly, Huang et al. highlighted the importance of timing antibiotic administration on treatment efficacy, as cancer patients who received antibiotics one month before ICI treatment had a higher hazard ratio than those receiving antibiotics two months before (HR = 2.23 versus 1.97)^20^. This might be explained by the partial recovery of the microbiota following antibiotic use^20^. Moreover, when antibiotics are administered before and during ICI treatment, it is associated with more symptomatic microbiotoxicity, such as diarrhoea, colitis, constipation, and other gastrointestinal effects^22–24^. While further work is needed, this association between antibiotic-induced gut dysbiosis and lower survival rates in ICI-treated cancer patients is concerning, particularly given the increased healthcare costs relating to patient hospitalisation and intensive care^25^.

The gut microbiome, especially in early life, has also been linked to vaccine efficacy^26^. Indeed, the development of the infant microbiome is associated with maturation of adaptive immunity and lymphoid tissue architecture. In a study of 48 Bangladeshi infants, the presence of keystone *Actinobacteria*, especially *Bifidobacterium* species, which are known for their crucial role in supporting immune system and maintaining healthy microbiomes, was associated with more robust immune responses to vaccines against polio, tetanus, and tuberculosis^27^. Given that 80% of children under the age of two are prescribed antibiotics, known to impact the gut microbiome, there is a need to investigate how this impact may influence vaccine efficacy during this developmental window^4,5^. While antibiotics remain indispensable for managing bacterial infections in early childhood, a deeper understanding of host-microbiome interactions could guide the development and more effective clinical use of antibiotics with lower microbiotoxicity or more targeted effects, thereby preserving immunological integrity and supporting improved health outcomes.

Considering the apparent relationship between the microbiome and the outcome of clinical interventions, such as cancer immunotherapy and vaccines, it has been proposed that the microbiome could be selectively modified to improve the efficacy of the interventions^26,28^. Avenues for this modulation include selective use of antibiotics, probiotics (live microorganisms), prebiotics (compounds that promote beneficial microbes), and faecal microbiota transplant (FMT).

For example, bone marrow transplant recipients are severely immunosuppressed and consequently at an increased risk of serious infections. Prophylactic antibiotics are often used to minimise this risk, but growing evidence shows that antibiotic-induced microbiome disruption in this group is associated with a significantly increased risk of developing graft-versus-host disease (GVHD)–a condition where the donor’s immune cells attack the recipient’s body^29–32^. Notably, overgrowth of *Enterococcus* species has been linked to GVHD severity and poor outcomes, underscoring the importance of maintaining microbial balance during treatment^33^.

FMT has emerged as a promising strategy to restore microbiome integrity post-transplantation. Studies using donor or autologous FMT, a patient’s own pre-treatment stool, have demonstrated that FMT can accelerate microbiome recovery and improve clinical outcomes in post-donor stem cell transplant patients^34,35^. These findings highlight the microbiome’s significant immunological role and its potential as a therapeutic target in transplant medicine. However, such strategies require further research in larger trials to minimise the possibility of inter-patient variability and ensure that interventions do not impact host microbial diversity or accelerate the spread of antibiotic resistance genes.

These findings underscore the importance of considering the effect of gut microbiome in non-microbiome therapies and may be relevant to prescribing practices to enhance subsequent treatment efficacy and reduce drug toxicity. This approach aligns with AMR stewardship aims, including optimising antibiotic use, reducing resistance, protecting public health, and reducing the economic burden associated with AMR^36^.

## Microbiotoxic properties of non-antibiotic drugs

While the microbiotoxic effects of antibiotics and their influence on treatment outcomes are well recognised, growing evidence suggests that non-antibiotic drugs can have a similar impact. Emerging evidence indicates that, similar to antibiotics, non-antibiotic drugs can disrupt the gut microbiome, leading to changes in both the efficacy and toxicity of subsequent therapies^37^. Recent studies further indicate that, beyond their microbiotoxic effects, certain non-antibiotic drugs can also facilitate the horizontal transfer of antibiotic resistance genes within microbial communities, underscoring an additional and often overlooked pathway by which these agents may contribute to the spread of antimicrobial resistance^38^. These effects have been reported with different drug classes, including nonsteroidal anti-inflammatory drugs, antidepressants, statins, etc.^39–42^. Here, examples of microbiotoxicity associated with non-antibiotic drugs are provided, focused on antisecretory drugs, due to their frequent clinical co-prescription with antibiotics and other medications, antipsychotics, which are increasingly prescribed worldwide, and diabetes treatments such as metformin, a mainstay therapy used globally. These examples demonstrate the breadth and impact of these unintended effects of drugs on health and patient outcomes.

### Antisecretory drugs

A notable example is demonstrated through the impact of antisecretory drugs, such as proton pump inhibitors (PPIs) and potassium-competitive acid blockers (P-CABs), on patient health outcomes. In a study of patients with metastatic urothelial carcinoma, those who took PPIs or P-CABs within 30 days before starting pembrolizumab treatment, experienced significantly shorter progression-free survival (P-CABs, *p* = 0.005; PPIs, *p* = 0.001), and, in the case of P-CABS, shorter overall survival (*p* = 0.018) compared to patients not taking these drugs^43^. The study suggests that antisecretory drugs may compromise cancer treatment effectiveness by altering the gut microbiome^43^. Additionally, PPIs have been associated with an increased risk of enteric infections due to *Clostridioides difficile* and *Shigella* species, and decreased microbiome diversity, further highlighting their potential to disrupt the gut microbiome^44^. Despite PPIs and P-CABs being highly prescribed worldwide, research into the microbiome implications of these and other antisecretory drugs remains sparse in the literature. This is a pressing concern, especially given the implications for polypharmacy and the specific vulnerabilities of demographics frequently on multiple medications, such as the elderly^45^.

### Antipsychotics

Antipsychotics have also been shown to alter the relative abundance of bacterial species linked with metabolic disorders and gastrointestinal effects^39,46–48^. Bhar et al. found that long-term ( > 12 months) risperidone use in male children and adolescents is linked with gut microbiome patterns that are associated with obesity, specifically, a significant decrease in the *Bacteroidetes:Firmicutes* ratio compared with healthy individuals (*p* < 0.05)^48^. Chait et al. demonstrated through an ex vivo human colon model that aripiprazole, a common antipsychotic drug, significantly inhibits gut microbiome growth, causing microbiome disruption, and reducing the proportion of *Firmicutes* and *Actinobacteria*^49^. Furthermore, recent evidence has shown that even in drug-naïve patients, the administration of antipsychotics is associated with metabolic dysregulation and weight gain^50^. Although evidence showing a causal dysbiotic effect of antipsychotics in humans is limited, these findings suggest antipsychotic-microbiome interactions that warrant further investigation, especially given the increasing prescription of antipsychotics to children and adolescents globally^46,47^. This could help improve treatment efficacy and reduce antipsychotic side effects, such as obesity and weight gain.

### Diabetes medication

Metformin, a common treatment for type 2 diabetes (T2D) worldwide, has also been shown to decrease gut microbial diversity and increase the abundance of opportunistic pathogens^51–55^. For instance, a three-month course of metformin in treatment-naïve T2D patients led to gut microbial changes associated with metabolic disorders such as obesity and irritable bowel syndrome^10,51^. Additionally, the study also reported an increase in *Escherichia-Shigella* species with metformin treatment, which are known to be associated with gastrointestinal symptoms like diarrhoea and bloating^51,53,55^. Notably, recent research indicates that metformin may also promote the enrichment of multidrug resistance genes in the gut microbiome of T2D patients, potentially through its effects on Escherichia^56^. These findings suggest that microbiotoxicity may be an important factor in clinical care to optimise therapeutic outcomes while preserving microbiome health. Furthermore, a study by Nakajima et al. involving 21 T2D participants demonstrated possible microbiotoxicity effects, as metformin-treated patients experienced increased regurgitation, diarrhoea, and abdominal symptoms associated with specific gut microbial disruptions, including increases in genera such as Tyzzerella, which has been linked to a higher risk of cardiovascular disease^53^. Although mechanistic data remain limited, the apparent microbiotoxic effects of metformin warrant further research, especially given its increasing use beyond diabetes, in conditions like polycystic ovary syndrome and certain types of cancer^54^. Improved understanding could provide insights for more effective prescribing practices and improved patients’ health outcomes.

These findings indicate that, like antibiotics, non-antibiotic drugs also display microbiotoxic properties, including disruption of microbial diversity, increased susceptibility to infections, and metabolic dysregulation linked to diseases like obesity. Similarly, non-antibiotic drugs also appear to affect the efficacy and toxicity of concurrent and downstream interventions. These findings highlight the need for further research, which may inform greater consideration of microbiotoxicity by drug developers and prescribers, to improve treatment efficacy and reduce toxicity, chronic diseases, and healthcare costs.

## Moving forward

By reviewing published evidence of the interplay between the microbiome and medicinal drug products, this paper highlights the clinical value of developing treatment approaches that prioritise preserving patients’ microbiomes, particularly by minimising the impact of microbiotoxicity. This new clinical approach offers a dual benefit- enhancing patient treatment outcomes whilst minimising the spread of AMR. Despite current limitations relating to the lack of standardisation in microbiome analysis, models, and biomarkers of toxicity, it is essential to build a reliable and meaningful knowledge base of drug-microbiome interactions for existing and novel drugs, through further multi-omics analyses and the inclusion of gut microbiome considerations in clinical trials^57–60^.

Research into measurements of microbiotoxicity in drug development pipelines, such as assessing the antimicrobial properties displayed by medicinal products on a patient’s microbiome, could advance our understanding of how different drugs interact with gut microbial communities^57^. Building this knowledge base could enable future drug development pipelines that use artificial intelligence to predict how treatments impact each patient’s microbiome, allowing for optimisation of therapeutic efficacy, reducing side effects, and ultimately improving overall health outcomes.

While we work on strengthening the microbiome analysis methods, we can begin to incorporate microbiotoxicity measurements into existing pipelines as a risk assessment or exploratory exercise to build the knowledge base. An example of an initiative that includes microbiome considerations is the UK Antimicrobial Products Subscription Model, created by the National Institute for Health Care and Excellence (NICE) and National Health Service England (NHS)^61^. The model delinks the volume of prescribed drugs from compensation and explicitly incentivises microbiome considerations in the development of novel antibiotics^61^. Antibiotics that meet the “Impact to microbiome” criterion are eligible for higher reimbursement fees^61^. Moving forward, building on the existing knowledge base can allow us to understand how microbiotoxicity evidence could inform current definitions of toxicity frameworks or contribute to resources that are readily accessible by healthcare providers, such as the summary of product characteristics and prescriber formularies.

The benefits of tackling microbiotoxicity may extend beyond patient health and AMR stewardship, as broader healthcare costs associated with microbiotoxicity remain uncertain. By tackling these gaps and building our knowledge base in microbiotoxicity and microbiome-drug interactions, we may enhance our capacity to produce and use stronger evidence to make effective treatment decisions, reducing the economic burden of AMR to the healthcare system and promoting microbiome and immune resilience. Microbiome considerations are increasingly relevant in drug development and AMR stewardship, with implications for the AMR landscape, drug efficacy and toxicity, and patient outcomes.

## Data Availability

No datasets were generated or analysed during the current study.
